# Familial Hypercholesterolemia in the Elderly: An Analysis of Clinical Profile and Atherosclerotic Cardiovascular Disease Burden from the Hellas-FH Registry

**DOI:** 10.3390/biomedicines12010231

**Published:** 2024-01-19

**Authors:** Christina Antza, Christos V. Rizos, Vasileios Kotsis, George Liamis, Ioannis Skoumas, Loukianos Rallidis, Anastasia Garoufi, Genovefa Kolovou, Konstantinos Tziomalos, Emmanouil Skalidis, George Sfikas, Michalis Doumas, Vaia Lambadiari, Panagiotis Anagnostis, Kimon Stamatelopoulos, Georgia Anastasiou, Iosif Koutagiar, Estela Kiouri, Vana Kolovou, Georgios Polychronopoulos, Evangelos Zacharis, Charalambos Koumaras, Chrysoula Boutari, Haralampos Milionis, Evangelos Liberopoulos

**Affiliations:** 1 3rd Department of Internal Medicine, Papageorgiou General Hospital, Medical School, Aristotle University of Thessaloniki, 54124 Thessaloniki, Greecevkotsis@auth.gr (V.K.); 2 Department of Internal Medicine, Medical School, University of Ioannina, 45110 Ioannina, Greecegliamis1@gmail.com (G.L.); anastgeorgia@hotmail.com (G.A.);; 3 Cardiology Clinic, Hippokration General Hospital, 54643 Athens, Greecepepescutajar@hotmail.com (I.K.); 4 Department of Cardiology, Attikon University General Hospital, Medical School, National and Kapodistrian University of Athens, 11527 Athens, Greece; lrallidis@gmail.com (L.R.);; 5 Second Department of Pediatrics, General Children’s Hospital “P. & A. Kyriakou”, Medical School, National and Kapodistrian University of Athens, 15452 Athens, Greece; garoufi@yahoo.gr; 6 Cardiometabolic Center, Lipid Clinic, LA Apheresis Unit, Metropolitan Hospital, 15562 Athens, Greece; genovefa@kolovou.com (G.K.);; 7 1st Propedeutic Department of Internal Medicine, AHEPA Hospital, Medical School, Aristotle University of Thessaloniki, 54636 Thessaloniki, Greece; ktziomalos@yahoo.com (K.T.);; 8 Cardiology Clinic, University General Hospital of Heraklion, 70013 Heraklion, Greece; 9 Department of Internal Medicine, 424 General Military Training Hospital, 56429 Thessaloniki, Greecebkoumaras@hotmail.com (C.K.); 10 Department of Internal Medicine, Hippokration General Hospital, Medical School, Aristotle University of Thessaloniki, 54642 Thessaloniki, Greece; 11 2nd Propaedeutic Internal Medicine Department, Diabetes Research Unit, Attikon University General Hospital, National and Kapodistrian University of Athens, 12462 Athens, Greece; 12 Department of Endocrinology, Police Medical Centre, 54627 Thessaloniki, Greece; pan.anagnostis@gmail.com; 13 Department of Clinical Therapeutics, Medical School, National and Kapodistrian University of Athens, 11528 Athens, Greece; stamatelopoulosk@yahoo.gr; 14 1st Propaedeutic Department of Medicine, Laiko Hospital, School of Medicine, National and Kapodistrian University of Athens, 15772 Athens, Greece

**Keywords:** familial hypercholesterolemia, dyslipidemia, elderlies, older age, HELLAS-FH registry, atherosclerotic cardiovascular disease

## Abstract

Background: Familial hypercholesterolemia (FH) carries a high risk of atherosclerotic cardiovascular disease (ASCVD). As the population ages, the age-related influence on clinical characteristics and outcomes becomes increasingly pertinent. This cross-sectional analysis from the HELLAS-FH registry aims to explore potential differences in clinical characteristics, treatment, ASCVD, and goal achievement between those younger and older than 65 years with FH. Results: A total of 2273 adults with heterozygous FH (51.4% males) were studied. Elderly FH patients (*n* = 349) had a higher prevalence of ASCVD risk factors, such as hypertension (52.1% vs. 20.9%, *p* < 0.05) and type 2 diabetes (16.9% vs. 6.0%, *p* < 0.05), compared to younger patients (*n* = 1924). They also had a higher prevalence of established ASCVD (38.4% vs. 23.1%, *p* < 0.001), particularly CAD (33.0% vs. 20.2%, *p* < 0.001), even after adjusting for major ASCVD risk factors. Elderly patients were more frequently and intensively receiving lipid-lowering treatment than younger ones. Although post-treatment LDL-C levels were lower in elderly than younger patients (125 vs. 146 mg/dL, *p* < 0.05), both groups had similar attainment of the LDL-C target (3.7% vs. 3.0%). Conclusions: Elderly FH patients have a higher prevalence of ASCVD, particularly CAD. Despite more aggressive treatment, the achievement of LDL-C targets remains very poor. These results emphasize the importance of early FH diagnosis and treatment in reducing ASCVD.

## 1. Introduction

Familial hypercholesterolemia (FH) is a condition affecting over 25 million people worldwide, characterized by severely elevated levels of low-density lipoprotein cholesterol (LDL-C) and increased atherosclerotic cardiovascular (CV) disease, morbidity, and mortality, often at an early age. Increased levels of LDL-C remain—per se—one of the major independent cardiovascular risk factors mainly due to an acceleration of the normally expected atherosclerotic procedure [1,2]. Vice versa, decreasing the LDL-C levels seems to lead to a reduction in atherosclerotic events, with even a reported 10% decrease from the first year. Importantly enough, a lifelong reduction in LDL-C would be expected to reduce atherosclerotic CV events by almost 50% per every m/l decrease in LDL-C [3]. This is of major importance if we take into account the scenario of FH, which leads to an almost 20 times higher risk of atherosclerotic CV disease [4,5].

However, FH is underdiagnosed, with less than 1% of cases identified in most countries [6]. Data from the global FH registry indicate that diagnosis is frequently delayed, with a median diagnosis age of 44.4 years, and even then, the LDL-C target is still not achieved [7]. This is due to the fact that FH patients either do not receive therapy or receive single-drug therapy, living with even 3 times higher LDL-C levels compared to the cut-off value recommended by guidelines [2,7]. So far, evidence is lacking regarding the percentage of underdiagnosis and undertreatment in the elderlies, in which, usually, the awareness for reducing the CV risk factors is increased, and evidence is also lacking as to whether this lack of appropriate treatment affects the CV profile of these patients later in life. 

The FH population has shifted towards the elderly as the global population ages and modern lipid-lowering treatments reduce atherosclerotic CV mortality. Indeed, 21.9% of FH patients in the global FH registry were over 60 years old [7]. Older age is an independent risk factor for atherosclerotic CV disease and is often accompanied by additional risk factors. Scant data are available for elderly FH patients regarding risk factor profile, atherosclerotic CV disease burden, and management status [7]. The unique challenges posed by aging, such as increased comorbidities and changes in metabolism, make it particularly important to understand how FH manifests and should be treated in older patients. This is especially relevant in light of the global trend of increasing life expectancy and consequent burgeoning of the older population. Moreover, the physiology of aging presents unique challenges in the management of chronic diseases like FH, including a distinct lipid profile and possible variations in response to treatment.

Hence, this study aimed to investigate clinical characteristics, lipid profile, treatment intensity, and atherosclerotic CV disease prevalence in elderly FH patients compared to younger ones in the HELLAS-FH registry. 

## 2. Materials and Methods

### 2.1. Study Design

The HELLAS-FH registry was established by the Hellenic Atherosclerosis Society as a national, multicenter registry to identify FH patients all over Greece and record treatment and clinical outcomes on an annual basis. The protocol was approved by the local ethical committee of each participating site as well as the Hellenic Data Protection Authority. The design and rationale of the HELLAS-FH registry have been previously described [8,9]. Patients included in the registry are those with a possible, probable, or definite diagnosis of FH, based on the Dutch Lipid Clinic Network (DLCN) criteria. Studies show that the DLCN criteria offer almost 85% agreement with the genetic test for FH [10]. Individuals either genetically diagnosed with homozygous FH or with baseline LDL-C ≥ 500 mg/dL, as well as patients < 18 years old, were excluded from the present analysis. The decision to prescribe lipid-lowering treatment was based on the 2019 European Society of Cardiology (ESC)/European Atherosclerosis Society (EAS) guidelines for the management of dyslipidemias, while therapy included any type of statins, ezetimibe, proprotein convertase subtilisin/kexin type 9 (PCSK9) inhibitors, n3 fatty acids, fibrates, bile acid sequestrants, or any possible combination of them [11]. The stratification of statin regimens was based on their LDL-C reduction efficacy: low intensity was delineated by an LDL-C decrement of less than 30%, moderate intensity by one of 30% to 50%, and high intensity was defined as achieving an LDL-C reduction of 50% or higher.

Laboratory exams were all performed in Greek public hospital laboratories. Medical history was recorded based on patients’ reports and confirmed by the national Greek prescription system, when applicable. The diagnosis of atherosclerotic CV disease included established atherosclerotic disease (either clinical or unequivocal on imaging), such as acute coronary syndrome, stable angina, coronary revascularization, stroke, and transient ischemic attack, as well as peripheral artery disease. Premature atherosclerotic CV disease was defined as events occurring in men under 55 or women under 60 years old. Patients were categorized as younger or older than 65 years old. Finally, the definition of hypertension and diabetes mellitus was based on relevant guidelines or history of receiving treatment for each disease, respectively [12,13].

### 2.2. Statistical Analysis

Continuous variables were tested for lack of normality by the Kolmogorov–Smirnov test. Values are expressed as mean ± standard deviation (SD) and median (interquartile range) for variables with and without normal distribution, respectively. Categorical variables are presented as frequencies. Categorical variables were compared using the chi-square test, and continuous variables were compared using the *t*-test or the Mann–Whitney U test depending on the variable distribution. In the logistic regression model, we chose to categorize age as a binary variable (younger than 65 years versus 65 years and older) to align with the primary objective of our manuscript, which was to compare these two distinct age groups. We included age only as a categorical variable and did not also use age as a continuous variable to avoid introducing multicollinearity. A *p* < 0.05 was considered significant. Analyses were performed using the Statistical Package for the Social Sciences (SPSS) 21.0 (SPSS Inc., Chicago, IL, USA).

## 3. Results

### 3.1. Baselines Characteristics

**Figure 1:**
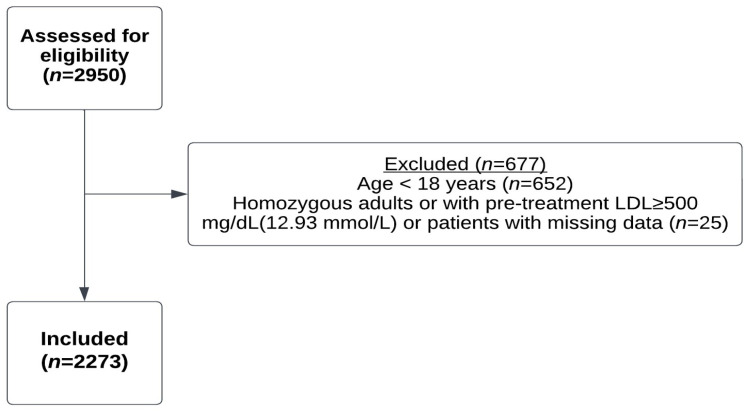
Flow chart of study population selection.

From a total of 2950 registry patients, 652 children were excluded, and an additional 25 patients with either missing data, homozygous FH, or LDL-C ≥ 500 mg/dL were also excluded (Figure 1). 

The study analyzed 2273 heterozygous FH adults, with baseline characteristics stratified by age presented in Table 1. Of the patients, 51.7% were male and 84.6% were younger than 65 years old, with a median age of 47 years for younger patients and 70 years for elderly patients. Male sex was more common among younger patients (53.2%) compared to older patients (43.6%) (*p* < 0.05 between groups). Patients aged 65 and older at the time of enrolment had been first diagnosed with FH later in life, with a median diagnosis age of 65 years. In contrast, the younger cohort, comprising individuals under 65 at the time of enrolment, had a median diagnosis age of 42 years. Elderly patients had a higher prevalence of hypertension (52.1% vs. 20.9%; *p* < 0.05) and type 2 diabetes (16.9% vs. 6.0%; *p* < 0.05), a lower frequency of smoking (24.6% vs. 40.1%; *p* < 0.05), and a higher body mass index (BMI) (27.4 vs. 26.7 kg/m^2^, *p* < 0.05).

### 3.2. Lipid Profile and Hypolipidemic Therapy 

Among the study patients, 65% (*n* = 1479) were already receiving treatment at registry enrolment, while the rest were not receiving any hypolipidemic treatment. The lipid-lowering treatment data among treated patients, stratified by age, is presented in Table 2. 

Younger patients had a lower treatment rate (63%) than older patients (75%; *p* < 0.05). Statin intensity did not differ between the two groups, but ezetimibe use was more common in older individuals than younger ones (54.4 vs. 45.7%; *p* < 0.05), as well as the use of ezetimibe–statins combination therapy (45.1% vs. 52.4%). There were no differences between the two groups as regards the other types of hypolipidemic therapy. 

**Figure 2:**
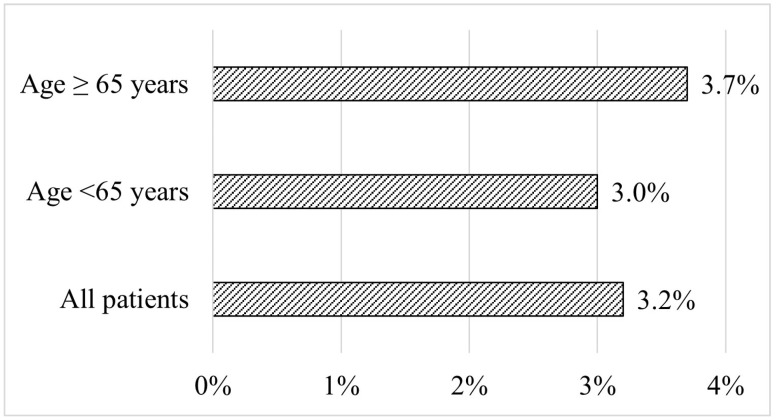
LDL-C target achievement stratified by age.

The effect of lower rates of treatment in the population aged less than 65 years, as well as the lower use of ezetimibe and combination therapy, is clearly depictured at Table 3, in which the lipid profile of patients before and after treatment is presented stratified by age. Before treatment, the two groups did not differ in terms of total cholesterol, high-density lipoprotein cholesterol (HDL-C), non-HDL-C, and LDL-C levels, except for triglyceride levels, which were higher in older patients compared to younger patients (141 vs. 128 mg/dL; *p* < 0.05). On treatment, LDL-C levels were lower in elderly than younger patients (125 vs. 146 mg/dL, respectively; *p* < 0.05) (Table 3). However, LDL-C target achievement was similarly very low in both groups: 3.7% in elderly vs. 3.0% in younger patients (Figure 2).

### 3.3. Atherosclerotic Cardiovascular Disease Burden

Data presented in Table 4 highlight the unadjusted differences in atherosclerotic CV disease burden between the two age groups. Elderly patients had a significantly higher prevalence of established atherosclerotic CV disease (38.4% vs. 23.1%) and premature atherosclerotic CV disease (30.1% vs. 20.9%), particularly coronary artery disease, stroke, and peripheral artery disease (*p* < 0.001 between groups). 

**Figure 3:**
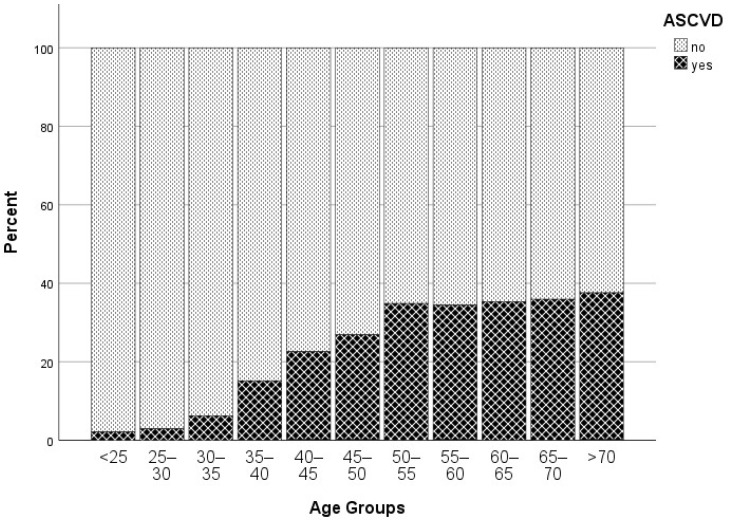
Stacked bars of patient percentage stratified by age and the presence of ASCVD.

**Figure 4:**
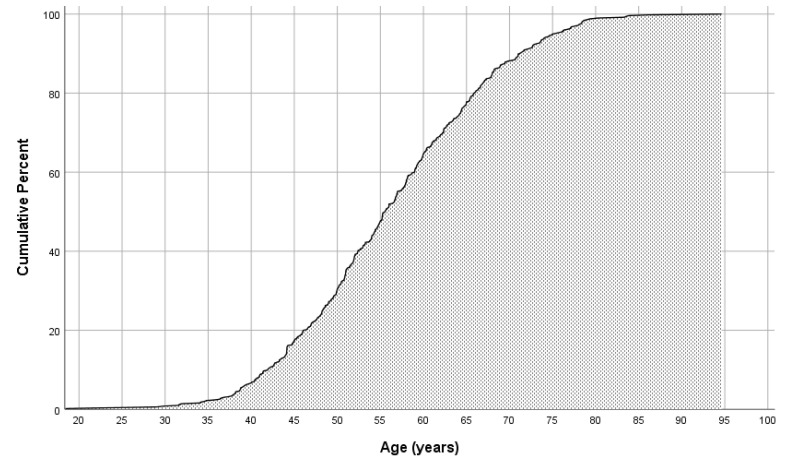
Cumulative percent of cardiovascular disease presence across ages among the subgroup of patients with established atherosclerotic cardiovascular disease.

The prevalence of atherosclerotic CV disease gradually increased with age, peaking at ages over 70 years old (Figure 3). In individuals with FH and established CV disease, the onset occurred at a young age, as early as 30–35 years (Figure 4). Approximately one-third of individuals with FH with atherosclerotic CV disease experienced the first event by the age of 50 years. Thereafter, atherosclerotic CV disease incidence remained stable until at least the age of 70 years. The incidence of new atherosclerotic CV events continued to rise beyond this age, albeit at a slower pace.

Logistic regression was conducted to assess the impact of age (older vs. younger) on prevalent atherosclerotic CV disease risk, adjusting for gender, hypertension, type 2 diabetes, smoking history, BMI, total cholesterol, TG, HDL-C, and LDL-C levels. The overall model was significant compared to the null model (*p* < 0.001), explained 32.7% of the variance in atherosclerotic CV disease (Nagelkerke R^2^), and accurately predicted 75.3% of cases. Elderly patients were more likely to have established atherosclerotic CV disease [OR 1.47 (95% CI 1.06–2.05), *p* = 0.023]. Male patients had twice the odds of having atherosclerotic CV disease than females [OR 2.51 (95% CI 1.92–3.29), *p* < 0.001]. The presence of type 2 diabetes (*p* = 0.001), smoking (*p* = 0.03), hypertension (*p* < 0.001), and lower HDL-C levels (*p* = 0.001) was also related to increased odds of established atherosclerotic CV disease. The remaining variables were not significantly associated with established atherosclerotic CV disease independently of other known CV risk factors.

Another logistic regression analysis was conducted to discern whether age at FH diagnosis affected the prevalence of atherosclerotic CV disease among patients with FH. The model included age at diagnosis; gender; hypertension; type 2 diabetes; smoking history; BMI; and levels of total cholesterol, TG, HDL-C and LDL-C. The overall model was significant compared to the null model (*p* < 0.001), explained 26.6% of the variance in atherosclerotic CV disease (Nagelkerke R^2^), and accurately predicted 78.8% of cases. Hypertension emerged as the strongest predictor (*p* < 0.001), followed by gender, with male patients being significantly more likely to have established atherosclerotic CV disease than females (OR 2.60, 95% CI: 1.98–3.43, *p* < 0.001). In addition, each year delay in FH diagnosis was associated with a 1.2% significant increase in the odds of established atherosclerotic CV disease (OR = 1.012, *p* = 0.008). The presence of type 2 diabetes (*p* = 0.001), smoking history (*p* = 0.03), and low HDL-C levels (*p* < 0.001) were also related to increased odds of established atherosclerotic CV disease. 

## 4. Discussion

The current study showed that older patients with FH exhibit a greater prevalence of atherosclerotic CV disease risk factors in comparison to younger ones. Elderly patients have a higher prevalence of established atherosclerotic CV disease (by 47%), even after adjusting for major atherosclerotic CV disease risk factors, indicating that aging seems to play an independent role in this regard. Elderly subjects receive lipid-lowering treatment more often and with greater intensity, particularly with more use of ezetimibe, compared with the younger subgroup. Nevertheless, both age groups fall short in achieving the recommended by guidelines LDL-C targets. 

The elderly subgroup had a lower percentage of male patients compared to the younger subgroup, which is consistent with existing data for older patients with FH [14]. One possible explanation for this finding could be the difference in atherosclerotic CV disease prevalence between genders, with male sex being an independent predictor of atherosclerotic CV disease in FH patients [15]. Therefore, the lower proportion of men in older age groups could be attributed to a potentially lower survival rate among male FH patients. Furthermore, lifestyle factors and gender-specific health behaviors might contribute to these differences; men may have higher rates of certain behaviors that increase cardiovascular risk, such as smoking or less healthful diets, which could result in earlier mortality. These multifactorial elements underscore the complexity of gender dynamics in FH and necessitate a gender-sensitive approach to treatment and prevention strategies.

In our study, we observed a lower prevalence of smoking among the elderly population, which raises several important considerations. Firstly, age-related changes in health behaviors, including increased smoking cessation in older age due to health concerns or medical advice, may have contributed to this difference. Additionally, survivor bias must be considered, as smokers may not survive to older age, especially in an inherently high-ASCVD-risk population like FH. Moreover, as public awareness of the health risks associated with smoking have increased and social norms around smoking have evolved, many long-term smokers may have quit.

Another noteworthy finding is the delayed diagnosis of FH, which was reported at 44 years old for the entire study population, which is in line with the existing literature [7]. However, the elderly patients had an even more delayed diagnosis of FH compared to younger patients (65 vs. 42 years). It can be postulated that elderly FH patients experienced delayed diagnosis due to low awareness of the disease and limited therapeutic options in the past. 

**Figure A1:**
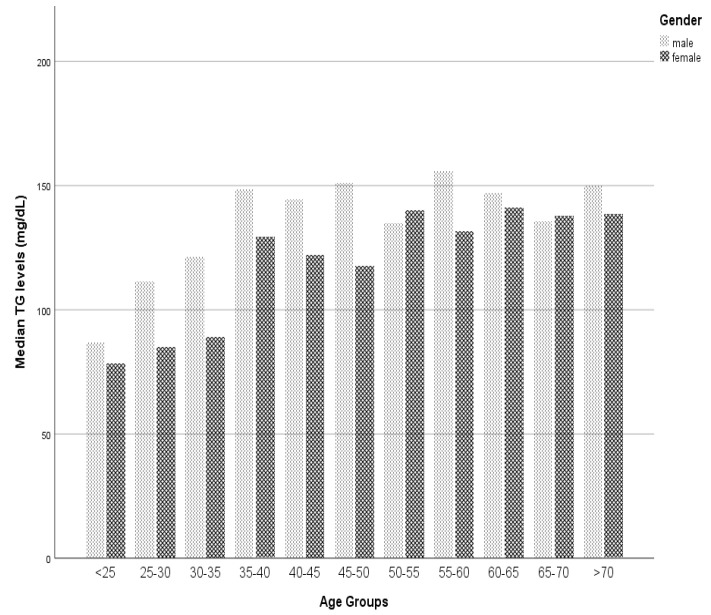
Median triglyceride levels by age group.

The prevalence of atherosclerotic CV disease risk factors, such as hypertension, dyslipidemia, and diabetes, increases with age [16,17]. This is confirmed in this study, where the elderly had a higher incidence of atherosclerotic CV disease risk factors. Of note, the elderly patients exhibited a higher pre-treatment TG. Indeed, an escalation in triglyceride levels was noted across both genders (Figure A1). This observation aligns with the existing literature in the general population [18,19]. Such a phenomenon could be potentially attributable to an array of physiological changes, including diminished postprandial triglyceride clearance, a reduction in lipoprotein lipase activity, an upsurge in ectopic fat deposition, as well as the higher rate of diabetes in the elderly [20]. 

When a long-life dyslipidemia exposure (FH) exists, a further increase in the risk is expected. Indeed, the risk of experiencing a fatal or non-fatal CV event is 50% for males and 30% for females by the age of 60 years old, if these patients stay untreated [21]. Even if this is already known, FH still seems to be a topic for which much more awareness is needed. In this study, 67% of FH patients received treatment upon registration, which is consistent with the global rate of 59.5% [7]. The percentage of FH patients receiving treatment was higher among the elderly group compared to the younger group. As expected, the elderly group had significantly lower on-treatment LDL-C levels compared to the younger group. To our knowledge, there is an evidence gap in the comparison of these age groups. Canadian FH adults older than 65 years old were found to receive satisfactory statin therapy (83% of those with definite FH) but did not remain on statins long-term, with the LDL target only being achieved in 10% of the definite FH population. However, there was no report for patients less than 65 years old [14]. Data from Finland showed that 96% of FH patients (>65 years old) were on lipid-lowering treatment—without specific data—with only 26% reaching the target, which is a much more satisfactory number compared to the one observed in our study. In patients < 65 years old, fewer patients were on therapy, but the target achievement was almost the same (with the exception of the group aged 24–44 years old reporting 30% achievement) [22]. 

As regards treatment, although the two groups had a similar frequency of statin use and intensity, the elderly group had a significantly higher use of ezetimibe. The use of mainly statins as a monotherapy, and less of other types of treatment (statins–ezetimibe combination therapy, PCSK9 inhibitors) has to be added as another crucial point on the observed high LDL levels. This observation is common with other studies in the field, where high-density statins are the preferable choice, escalation seems not to be followed, and LDL levels are elevated [7,23,24,25,26]. A large study from Spain (SAFEHEART Registry) tried to inform and educate physicians on dealing with FH patients, and after that, followed these patients for one year. In the baseline, only 43% of the treated population was receiving high-dose statins, 26% combination therapy, and 0.4% PCSK9 inhibitors. Even after doctors were made aware, 64% of the population was still only on high-dose statins, without always reaching the LDL goal [27]. 

Given the late diagnosis of FH and subsequent prolonged exposure to elevated LDL-C levels, along with the close association between aging and other atherosclerotic CV disease risk factors, it is not surprising that elderly patients have a high atherosclerotic CV disease burden. Importantly, the difference in atherosclerotic CV disease prevalence remained significant even after adjusting for concomitant major atherosclerotic CV disease risk factors. While our study demonstrates a higher prevalence of atherosclerotic CV disease in older FH patients compared with younger ones, it is important to contextualize these findings within the broader epidemiological trend where atherosclerotic CV disease prevalence generally increases with age. The results from the relevant logistic regression analysis in our study indicate that within the FH population, the timing of diagnosis is a significant factor that may influence disease prevalence. Our results underscore the importance of early diagnosis and initiation of treatment in FH patients, as each additional year of diagnosis delay was associated with a modest but significant 1.2% increase in the odds of established atherosclerotic CV disease. The conclusion drawn from our study is not merely reflective of the general trend of increased CV disease with advancing age but emphasizes the amplified risk that delayed diagnosis and treatment pose to individuals with FH. Thus, while acknowledging the inherent risk increase with age observed in the general population, our study provides evidence for a targeted approach regarding the early diagnosis and management of FH to prevent or delay the onset of atherosclerotic CV disease.

An independent determinant of atherosclerotic CV disease in FH patients has been shown to be age, as demonstrated by a large-scale cohort study [28]. A study conducted in Norway revealed that the standardized atherosclerotic CV mortality ratio, when compared with the Norwegian population, was highest among the age group of 20–39 years (8.03), followed by those aged 40–59 years (2.49), 60–69 years (1.72), and 70–79 years (1.08) [29]. A separate study reported a rise in the incidence of stroke among individuals with FH as they advanced in age [30]. Of note, a prospective study conducted in a Norwegian FH population showed that the incidence rate of coronary heart disease increased with age [31]. The cumulative exposure to hypercholesterolemia over the lifespan of an individual with FH contributes to the observed additive effect, although other factors may also play a role [18,19,20].

Certain limitations of the present study should be mentioned: First, this is a cross-sectional study, and as such cannot establish causation or incidence. Additionally, a survival bias may be present, as the study only includes patients who have survived to the point of registration. Another limitation is that atherosclerotic CV disease events were captured using physician reports rather than adjudication, which may introduce some measurement errors.

## 5. Conclusions

Elderly vs. younger individuals with FH have a 47% higher prevalence of atherosclerotic CV disease, even after adjusting for major atherosclerotic CV disease risk factors. This finding underscores the importance of the early diagnosis and treatment of FH for preventing atherosclerotic CV disease.

## Figures and Tables

**Table 1:** Baseline characteristics of adult patients with familial hypercholesterolemia stratified by age.

		Total	Age (Years)	
			<65	≥65
Number of patients		2273	1924	349
Gender (male/female)		1176/1176	1024/900 *	152/197 *^,§^
Age at registration (years)		50 ± 14	47 (39–55)	70 (67–75) ^§^
Age at diagnosis (years)		44 ± 16	42 (32–51)	65 (53–70) ^§^
^†^ Physical findings (%)		14.6	13.5	20.3 ^§^
Corneal arcus before age 45		6.4	5.6	10.9 ^§^
Xanthoma		5.2	4.8	7.1
Xanthelasma		6.1	5.8	8.0
Hypertension (%)		25.7	20.9	52.1 ^§^
Type 2 diabetes (%)		7.7	6.0	16.9 ^§^
BMI (kg/m^2^)		26.8 (24.2–29.6)	26.7 (24.1–29.6)	27.4 (24.8–29.6) ^§^
Systolic blood pressure (mmHg)		127 ± 14	126 ± 14	134 ± 13 ^§^
Diastolic blood pressure (mmHg)		78 ± 10	77 ± 10	78 ± 9
Heart rate (bpm)		74 ± 10	74 ± 10	72 ± 10 ^§^
Ever smoked (%)		37.7	40.1	24.4 ^§^
Increased Waist Circumference (%)	Males (>102 cm)	23.2	23.0	24.3
	Females (>88 cm)	41.6	40.2	47.7

* *p* < 0.05 between males and females among specific group, ^§^ *p* < 0.05 between groups, ^†^: xanthoma, xanthelasma, or corneal arcus. Continuous variables are expressed as mean ± standard deviation (SD) and median (interquartile range) for variables with and without normal distribution, respectively. Categorical variables are presented as frequencies.

**Table 2:** Lipid-lowering treatment of patients with familial hypercholesterolemia stratified by age.

Parameter		All Treated Patients	Age	
			<65	≥65
Number of patients (% of total group population)		1479 (65%)	1218 (63% ^§^)	261 (75%)
^+^ All statins (%)		96.4	96.4	96.6
^+^ Statin intensity (%)	Low	1.6	1.8	0.8
	Moderate	35.9	36.7	32.1
	High	62.5	61.5	67.1
^+^ Ezetimibe (%)		47.3	45.7	54.4 ^§^
^+^ PCSK9i (%)		5.4	4.9	7.7
^+^ n3 fatty acids (%)		1.9	2.1	1.1
^+^ Fibrates (%)		1.8	1.7	2.3
^+^ Bile Acid Sequestrants (%)		1.5	1.2	2.8

^+^ Among patients on treatment, ^§^ *p* < 0.05 between groups.

**Table 3:** Lipid profile of adults with familial hypercholesterolemia stratified by age.

	Pre-Treatment			Post-Treatment		
Parameter (mg/dL)	Total	Age		Total	Age	
		<65	≥65		<65	≥65
Total cholesterol	320 ± 64	319 ± 64	324 ± 66	219 ± 65	223 ± 67	203 ± 55 ^†^
Triglycerides	130 (95–180)	128 (94–180)	141 (102–185) ^†^	110 (79–152)	110 (80–152)	106 (78–151)
HDL-C	51 ± 16	51 ± 16	52 ± 15	51 ± 16	51 ± 16	53 ± 17 ^†^
non-HDL-C	268 ± 64	267 ± 63	271 ± 65	163 ± 59	166 ± 59	149 ± 55 ^†^
LDL-C	238 ± 61	238 ± 60	240 ± 62	142 ± 58	146 ± 59	125 ± 50 ^†^

HDL-C: high-density lipoprotein cholesterol, LDL-C: low-density lipoprotein cholesterol. Data are presented as mean ± standard deviation or median (interquartile range) for parametric and non-parametric variables, respectively. ^†^ *p* < 0.05 between groups.

**Table 4:** Unadjusted atherosclerotic cardiovascular disease burden of adult familial hypercholesterolemia patients stratified by age.

Parameter	All Patients	Age		*p* between Groups
		<65	≥65	
ASCVD (%)	25.4	23.1	38.4	<0.001
Premature ASCVD (%)	22.3	20.9	30.1	<0.001
CAD (%)	22.1	20.2	33.0	<0.001
Premature CAD (%)	19.8	18.5	26.6	<0.001
Stroke (%)	3.0	2.4	6.3	<0.001
PAD (%)	2.7	2.2	5.4	0.001

ASCVD: atherosclerotic cardiovascular disease, CAD: coronary artery disease, PAD: peripheral artery disease.

## Data Availability

Data are contained within the article.

## Abbreviations

LDL-C: low-density lipoprotein cholesterol, FH: familial hypercholesterolemia, CV: cardiovascular, DLCN: Dutch Lipid Clinic Network, PCSK9: proprotein convertase subtilisin/kexin type 9, BMI: body mass index, HDL-C: high-density lipoprotein cholesterol, Lp (a): lipoprotein (a), CAD: coronary artery disease, FHSC: Familial Hypercholesterolaemia Studies Collaboration.
